# Personal and professional challenges in the management of deliberate self-poisoning patients in rural Sri Lanka: a qualitative study of rural hospital doctors' experiences and perceptions

**DOI:** 10.1186/1471-2458-8-373

**Published:** 2008-10-28

**Authors:** Lalith Senarathna, Jon Adams, Dhammika De Silva, Nick A Buckley, Andrew H Dawson

**Affiliations:** 1 South Asian Clinical Toxicology Research Collaboration, Faculty of Medicine, University of Peradeniya, Peradeniya, Sri Lanka; 2 Discipline of Social Science, School of Population Health, University of Queensland, QLD, Australia; 3 Planning unit, Provincial Director of Health Services office, North Central Province, Sri Lanka; 4 POW Hospital Clinical School, University of NSW, Australia; 5 School of Population Health, University of Newcastle, Newcastle, Australia

## Abstract

**Background:**

Deliberate self-poisoning is a major public heath issue in developing countries. In rural Sri Lanka deliberate self-poisoning is one of the leading causes of hospital death. The majority of patients with poisoning present to rural hospitals for initial treatment that are staffed by non-specialist and often relatively junior doctors. The treatment of self-poisoning patients poses numerous clinical challenges and further difficulties are experienced if patients are uncooperative and aggressive, intoxicated with alcohol or suffering mental illness. Previous research in developed countries has examined self-poisoning patients and their treatment but little is know about self-poisoning patient care in the context of rural health provision in developing countries. This study provides the first focused exploration of the experiences and perceptions of primary care rural hospital doctors in Sri Lanka toward the treatment of self-poisoning patients.

**Methods:**

Semi-structured in-depth interviews were conducted with fifteen doctors from rural hospitals in the North Central Province, Sri Lanka. All interviews were recorded and transcribed and subject to thematic analysis.

**Results:**

Participating doctors did perceive that treating self-poisoning patients in a primary care rural hospital as potentially confidence-building. However, resource issues such as the lack of medication, equipment and staffing were seen as important challenges to treating self-poisoning patients. Other challenges identified included disparity with community and other staff members regarding expectations of care, a sense of professional isolation and a lack of continuing education programs.

**Conclusion:**

Addressing professional isolation through educational and trainee programs for doctors and reducing the variance in expectations between professional groups and the community has the potential to improve delivery of care for self-poisoning patients.

## Background

The high mortality and morbidity rates from deliberate self-poisoning are major heath service delivery issues in developing countries[1]. In rural Sri Lankan districts, deliberate self poisoning is one of the leading causes of hospital deaths[2,3]. The social and health care burden of self-poisoning has been recognized as a priority public health issue in Sri Lanka by the Presidential Working Group for Suicide Prevention and researchers have rightly identified improved medical management of such cases as a frontline strategy to reduce the number of deaths from self-poisoning in the developing world[4].

The treatment of self-poisoning poses numerous clinical challenges; these often severely poisoned patients can be medically unstable and require resuscitation, specific treatment and close observation and monitoring. Further difficulties in delivering care occur in patients who may be uncooperative and aggressive, intoxicated with alcohol, or mentally ill[5].

The majority of patients with poisoning present to rural hospitals for their primary treatment. These hospitals are staffed by non-specialist and often relatively junior doctors. The provision of appropriate treatment resources, such as antidotes, and resuscitation facilities to these primary care rural hospitals, together with adequate trained staff may reduce hospital mortality[6,7]. Nevertheless, the delivery of care is also significantly influenced by staff experience and perceptions. Hence exploring the perception and attitude of rural hospital doctors is important to improve the poisoning patient care in rural hospitals to reduce mortality from poisoning.

While previous studies have explored clinicians' and nurses' attitudes towards self-harm patients in well resourced tertiary centres there is no published research examining practitioners' experiences and perceptions regarding the care and treatment of self-poisoning patients in rural, low resource settings [8,9]. In response, this paper reports results from a study exploring the experiences and perceptions of primary care rural Sri Lankan hospital doctors towards treating self-poisoning patients.

## Methods

The project was approved by the Ethics Review Committee, Sri Lanka Medical Council (ER001/06) and the Human Research and Ethics Committee, University of Newcastle, Australia (H-217-0506).

**Figure 1:**
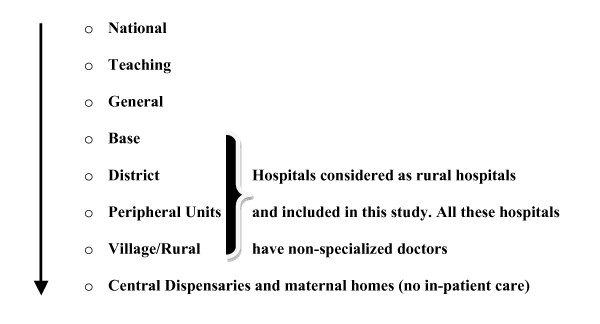
**Categorization of Sri Lankan hospitals**. Categorization of hospitals and place of each hospital in Sri Lankan health system according to the level of resources available in the hospital and for the hospital from health ministry budget. Study hospitals without specialised doctors are highlighted with brackets.

Semi-structured face-to-face interviews were conducted with primary care hospital doctors currently working in rural hospitals with in-patient facilities in the North Central Province of Sri Lanka. Although the selection of participants was undertaken on a voluntary basis, attempts were made to recruit informants with a range of demographics and categories of hospital practice settings. There are 56 rural hospitals situated in North Central Province. However, recruitment focused only upon those doctors practicing within 45 of these hospitals which provided in-patient facilities at the time of study (figure 1).

Invitations to participate in the study were sent to 40 hospitals (5 hospitals were excluded due to a deteriorating security situation in the immediate locale). Finally, doctors from 13 hospitals participated for the study,

Participants were provided with study information on common notice boards in the hospitals and by direct contact from the hospital in-charge officer. Twenty-eight doctors agreed to participate. Written consent was obtained only from participating doctors before conducting each interview. Interviews were around 20 – 30 minutes in duration and employed a semi-structured theme guide. The theme guide was loosely based around the exploration of three distinct yet interrelated domains of enquiry: the doctor's experiences and perceptions regarding the current treatment of self-poisoning patients; the doctor's understanding of how the rural hospital setting and any staff relations therein may influence and shape the current treatment of self-poisoning patients; and the doctor's perceptions of the challenge and future opportunities associated with treating self-poisoning patients in rural hospitals.

Interviews were undertaken until a point at which thematic saturation was achieved (where no new rich information is being elicited) – 15 interviews in the case of this fieldwork[10].

According to the Sri Lankan Ministry of Health statistics there are 3200 doctors working in secondary and primary hospitals at a provincial level, of these approximately 1000 doctors currently practice in rural hospitals. While not statistically representative of the wider population, an attempt was made to include a cross-section of participants in line with the general demographics of the wider doctor population working in Sri Lankan rural hospitals. Thirteen male and two female doctors participated in the interviews and ten of them were in the 35 – 40 age group. The experience of participating doctors ranged from one year to eight years; 13 doctors had more than two years experience.

Interviewees were encouraged to answer in their own words and any additional themes or concerns beyond the guide were also accommodated with open-ended questions. All interviews were conducted in Sinhalese (mother language of all participants), recorded and transcribed. Each transcribed interview was translated into English and the translations were independently validated individually by a physician and a translator. The transcribed interviews were then read, coded and analysed to extract themes. Thematic analysis was conducted concurrent to fieldwork and both data collection and analysis followed an inductive approach whereby investigation was refined and revised to fit the evolving data and findings. Two members of the research team provided independent coding and analysis to cross-check codes and themes and to develop an overall interpretation of the data. Analytical rigor was also enhanced by searching for negative cases in code and theme development[11].

## Results

Qualitative analysis of the interview data provided an exploration of the doctor's experiences and perceptions about treating self-poisoning patients. Four main themes were revealed from the analysis and are presented here with relevant quotes from the interviews.

### 1. Conceptualising self-poisoning as a health problem: common primary treatment or transfer for specialist care

The initial assessment of self-poisoning by rural hospital doctors determines their subsequent treatment plan and further investigations. The vast majority of respondents suggested that self poisoning patients should be approached and managed in a similar fashion to all other presenting patients:

"We do not consider poisoning as being a different sort of condition. It is just another emergency condition and we attend it in our usual capacity" [Dr08]

Other respondents provided similar claims;

"We need to identify patients who have really ingested dangerous amounts. Some patients are admitted after also ingesting alcohol and they have just spat out the poison and have little toxicity. Patients who have really ingested a dangerous amount of poison can be identified using clinical features and our experience. There are only a few patients with known or established psychiatric diagnosis. Patients normally accept treatment because they do not want to die." [Dr 04]

Self-poisoning is obviously an issue that requires additional attention and investigation quite apart from the 'normal' clinical response and the rural hospital doctors were keen to explain their sensitivities related to this issue. However, they do not see such additional factors (often presented as mental health issues) as necessarily their domain or as impinging significantly upon their obligation to provide treatment:

"We tend to think immediately of (medical) treatment, but patients have reasons to self poison which also need to be addressed. Sometimes the reason for self-harm is a mental illness or it can be a family or financial problem. As we often don't know the cause, these should not be considered as separate types of patients and we should not differentiate them from other patients. We are here to (medically) treat them; their other problems should be addressed separately" [Dr 02]

Meanwhile, others explained how certain patient types associated with self-poisoning are perceived as specially challenging cases:

"Most of the male poisoned patients are drunk and troublesome. Treating such a person is different from treating other emergency situations" [Dr 11]

As explained in the following section, the perception of such challenging behaviours and their detrimental effect upon clinical work and decision-making is not confined solely to the patient but can also extend to other staff and the wider community involvement associated with self poisoning patients.

### 2. Staff and Community Relations: behaviors and expectations

The interaction of rural hospital doctors with the hospital staff and the local community was seen to be important amongst the participants. The doctors explained how the behaviour and expectations of the staff and the community influenced the interaction and the treatment of self-poisoning and the tendency of other staff members to comment on the doctor's treatment of the self-poisoning was considered a barrier to optimal decision making.

The participants also explained how inappropriate behaviours and unreasonably high expectations on behalf of patients and their relatives produced a sense of extra pressure. Such circumstances were seen as often leading to more transfers to secondary hospitals resulting in greater use of resources even though many of these self-poisoning cases could have been managed in rural hospitals.

"You observe they [relatives and friends of the patient] come in large numbers and stay around the patient. Although they listen to me, the nurses cannot control them. With 50 people around you, it is not possible to observe the patient.

They always want to see something being done to the patient, not just someone waiting and observing" [Dr 08]

"A patient who is bitten by a snake normally comes in with a few people and they are cooperative. But poisoned patients are brought to hospital by large crowds and create problems with hospital staff. Such incidences make treating poisoned patients an unpleasant experience" [Dr 11]

"If the bystanders and relatives are a problem, I transfer the patient even if it is not really indicated. There are other patients to look after; I cannot waste my time to control a crowd" [Dr 01]

The challenge of friends' and relatives' behaviours and expectations are also seen by these doctors as occasionally spilling over into the ranks of other assisting hospital staff. As the following quote illustrates, the doctors are keen to stress the central importance of assisting staff in aiding or disrupting the clinical treatment and management of self-poisoning patients:

"If the other staff in my hospital see that more patients recover after my treatment, then they decide that I am a good doctor. That's how they think. Sometimes they praise or criticize doctors with villagers. Hospitals workers are usually from the same villages (as the patients) and they know each other. Therefore comments from other staff can make a rural hospital doctor's life either easier or more difficult. " [Dr 05]

Similarly, but in reverse, the rural hospital doctors also explain how the actions and reactions of other hospital staff (in the case below, doctors in secondary hospitals) to treatment decision-making can sometimes spark animosity and reflect badly upon the rural doctors in terms of patient, relatives and wider community sentiment towards their work:

"When we transfer patients, the doctor from the secondary hospital admission area sometimes asks for the reasons why we were so late in sending the patient to their hospital. When a patient or relative hears this, they probably think that we are poorly managing in rural hospitals and wasting time by just doing nothing" [Dr 06]

### 3. Resources and Locations: keeping up to date and in touch

Another group of issues described by the doctors regarding self-poisoning care revolved around the assigned resources and locations of their respective hospitals. The doctors describe how a serious lack of human and other resources in the rural hospital setting does not support the professional development of rural hospital doctors. These issues can affect not only the doctor's professional life but also their personal life and generate feelings of insecurity.

"In my first week here (in this hospital), I intubated a poisoned patient, sucked out the secretions (*This is the standard initial treatment for a patient who has ingested a poison like an organophosphate pesticide. Airways are secured with an inserted tube and secretions which block the airway removed using a sucker*) and transferred them to [a secondary hospital] in an ambulance with a nurse. The officer-in-charge was not happy and asked me not to do it regularly, as without nurses he cannot run the hospital. His suggestion was to transfer with an attendant without intubating. If that patient was transferred without a secure airway and without monitoring by a nurse, the patient probably would have died on the way" [Dr 06]

"Although we want to just observe some poisoned patients without sending them to secondary hospitals, there are no nurses to observe. I cannot sit here and observe patients because the outpatients will wait in a queue until I come. So we have no other option other than to transfer" [Dr 01]

"Sometimes we have to think twice before giving atropine (an antidote). Because all we have is a few vials, if we use them for moderate or less unwell patients, what will we do when a very ill patient comes?" [Dr 02]

Meanwhile, others interviewed pointed to the challenges of continuing professional education (CPE) in a rural hospital setting and how such challenges can limit the effectiveness of care for the self-poisoning patient:

"A few years back, a senior physician visited these hospitals and talked to us about the initial management of poisoned patients. After that most of us were interested in learning about toxicology. But nobody visited these hospitals after that time and there was no further interaction with experts" [Dr 05]

"It would be better if there was a way to receive information about new treatment methods and guidelines. I only have this (referring to copy of National Poisons centre book). This is not even the latest version" [Dr 07]

"There is no way for us to learn about new poisons and treatments. It is apparent that the pattern of poisoning has changed from our university years. The poisonings we learnt about in those days are not seen now. And if there was a workshop or training from the department of health or a clinical society, how could I close the hospital and go there? We are like frogs in a well" [Dr 10]

The challenge of continuing professional education could be seen as rendering care of self-poisonings in rural hospitals not only far from ideal but perhaps redundant leading to an inevitable transfer of self-poisoning patents to secondary hospitals (a prospect undermined by the earlier suggestion that nursing staff and other requirements for successful transfer may not always be available). As one doctor explains;

"I think the skills of rural hospital doctors are not sufficient to stabilize the condition of an unwell poisoned patient. That's why large number of patients are transferred to secondary hospitals. For example, if someone is not confident in making the airway safe in poisoned patients, then there is no option other than to transfer. That is the attitude" [Dr 04]

Likewise, another doctor explains how he perceives rural hospital doctors as lacking skills and training regarding successful treatment of respiratory failure – a common mode of death from some poison types:

"Every doctor working in rural hospitals should be given anesthetic training. If you cannot secure the airway when a patient cannot breathe properly, there is no point to working in a primary hospital" [Dr 10]

### 4. Experience improves focus and confidence in treating and/or transferring self-poisoned patients

Despite the challenges and limitations associated with the care of self-poisoning patients in rural hospitals, the doctors do also acknowledge the potential and opportunities available in their clinical setting for self poisoning patient care, successful treatment and appropriate assessment for the transfer of self-poisoning patients. Some interviewees highlight how the rural hospital setting provides a first 'port of call' for self-poisoning care and treatment:

"The X hospital [large urban hospital] where I worked previously, received only a few poisoned patients and most of these were transferred from rural hospitals. I used to check the patient's notes to get the patient's history and assess the severity. But here, I am the first person to see the patient. Therefore it is necessary to take more time on assessment and to do it properly." [Dr 02]

Other doctors linked such first line grass-roots exposure to an eventual gain in experience in treating self-poisoning – a development which is seen as providing greater physician confidence and, ultimately, delivering better organized and responsive treatment:

"When I was appointed to this hospital from a secondary hospital, I was afraid even to discuss pesticide poisoning cases such as paraquat. I did not know what to do or how to prepare for the situation. This was because all the people expected me to be able to do the best for the patient and I wasn't sufficiently confident. However, now (after 4 years) I feel OK; that I can manage the situation" [Dr 05]

And others explained how they had responded to the high incidence of self-poisoning by developing practices and processes specifically directed towards treatment and care of self-poisoning:

"I started a small unit (emergency unit) in a part of my office to keep our oxygen cylinder, sucker machine (we have one of each) and other emergency equipment and medicines. Now, when a patient is admitted, especially poisoned patients, there is no need to run here and there to collect everything. Emergency equipment is in the emergency unit and you can take the patient there after decontamination" [Dr 02]

## Discussion

Analysis of the study interview data identified four major themes important to helping understand the approach and practice of rural hospital doctors towards the treatment of self-poisoning patients. More specifically: the doctor's conceptualization of self-poisoning patients as similar to any other presenting emergency patient; the sometimes challenging relations with, and behaviours of, other hospital staff, patient relatives and others in the local community for the optimal treatment of self-poisoning patients; the difficulty of keeping abreast of treatment developments and the challenges facing continuing professional development in the topic of self-poisoning patient care in rural hospital settings; and the doctors' understanding that grass-roots exposure may eventually lead to improved care procedures and practices for self-poisoning patients in rural hospital settings.

The majority of participating doctors felt that self-poisoning patients should be treated the same as other emergency patients. But factors like alcohol ingestion and aggressive behaviours, and interference from family members and entrenched views of other hospital staff may alter these positive attitudes.

The doctors' experiences and perceptions highlight the lack of a system to regularly update knowledge about poisoning and emergency treatments. Although a national handbook of poisoning does exist and is available in virtually all hospitals in the areas studied, it is not clear how often it is used or if the guidelines are closely followed[12]. The national handbook has not been updated for several years, may not be user-friendly and sometimes fails to provide information relevant to grass-roots practitioners such as those interviewed in our study

Our findings also reveal how rural doctors perceive relations with other staff members as sometimes a barrier to optimal decision-making when treating self-poisoning patients. Rural hospital doctors and staff have more direct contacts with the community compared with those in secondary referral hospitals. It was apparent that the desired cooperation from the community was not always perceived as sufficient by the doctors. Also, unnecessary influence from the patient's relatives, the community and long-term hospital nursing staff or attendants can inappropriately affect treatment decisions. It would appear from the analysis of our interview data that reshaping the community's understanding of the rural hospital's role in the treatment of self-poisoning may be necessary to improve community cooperation.

The interviews with the doctors identified the importance of experience in providing these practitioners with a clear focus and confidence necessary to providing less inappropriate transfers and more successful treatments for self-poisoning patients in their hospitals. However, there are only a few experienced doctors in rural hospitals in the North Central Province of Sri Lanka and it is the policy of health authorities to appoint more junior people to rural centers. This situation may contribute to the difficulty of improving management of self-poisoning in rural hospitals.

Although the medical treatments for self-poisoning are based on the experiences and perceptions of rural hospital doctors, related issues are perhaps best addressed through a multidisciplinary team approach. Whyte et al described a model for the management of self-poisoning patients[13]. The suggested model was a multidisciplinary approach considering psychiatric perspectives, nursing perspectives and medical perspectives. Such an approach to address the themes revealed from this study might be useful in enhancing the quality of care of poisoned patients in rural primary care hospitals.

We must be mindful of the limitations of the study reported here. The study was exploratory in nature providing an initial investigation of this under-researched topic strictly from the perspective of rural hospital doctors in a particular North Central Province of Sri Lanka. It is important that further research complement this work by broadening the research focus to incorporate the perspectives and experiences of other key stakeholders involved in the treatment of self-poisoning patients. For example, future enquiry may explore the perceptions and understandings of patient's relatives, rural hospital nurses and reception staff amongst others with regard to the issue of self-poisoning patients and their treatment. The qualitative work reported in this paper may also provide useful insights for designing a larger survey based project. A national survey of rural hospital doctors (amongst others) as a means of establishing the prevalence of particular practices and approaches and the mapping of educational and equipment needs for the treatment of self-poisoning patients would also help us to answer a number of significant research questions beyond the scope of the study reported here.

## Conclusion

Doctors in rural primary hospitals often feel isolated, and their decisions regarding emergency treatment of self-poisoned patients are frequently influenced by pressure from nursing or attendant staff or family members, and not enough by a confident understanding of recommended treatments. Introducing educational and trainee programs for doctors and hospital staff may be effective in addressing professional isolation. In addition, promoting a multidisciplinary team approach may reduce the variance in expectations between professional groups and the community. Such an approach has the potential to improve the delivery of care of poisoned patients in rural primary care hospitals at relatively low cost compared with the more obvious remedies of supplying additional resources and staff.

## Authors' contributions

Lalith Senarathna designed this study, acted as principal researcher, collected and analyzed data and wrote the first draft of the paper. Jon Adams helped with study design, qualitative data analysis and paper writing. Dhammika de Silva helped with data collection and recruitment. Nick Buckley helped with study design and contributed to paper writing. Andrew Dawson also helped with study design, supervision, data collection and paper writing. All authors helped improve the study design and finalize paper writing.

## Pre-publication history

The pre-publication history for this paper can be accessed here:


